# Perceived Nexus Between Non-Invigilated Summative Assessment and Mental Health Difficulties: A Cross Sectional Studies

**DOI:** 10.1007/s10805-023-09472-w

**Published:** 2023-03-31

**Authors:** Amanda Graf, Esther Adama, Ebenezer Afrifa-Yamoah, Kwadwo Adusei-Asante

**Affiliations:** 1 School of Nursing and Midwifery, Edith Cowan University, 270 Joondalup Drive, Joondalup, WA 6024 Australia; 2 School of Science, Edith Cowan University, Perth, Australia; 3 School of Arts and Humanities, Edith Cowan University, Perth, Australia; 4 School of Nursing and Midwifery, University of Notre Dame University, Fremantle, Australia

**Keywords:** COVID-19, Non-invigilated, Examination, Assessments, Stress, Test anxiety

## Abstract

The COVID-19 pandemic rapidly led to changes in the mode of teaching, learning and assessments in most tertiary institutions worldwide. Notably, non-invigilated summative assessments became predominant. These changes heightened anxiety and depression, especially among individuals with less resilient coping mechanism. We explored the perceptions and experiences of mental health difficulties of students in tertiary education regarding non-invigilated alternative assessments in comparison to invigilated assessments. A pragmatic, mixed method cross sectional design was conducted online via Qualtrics. Thematic analysis of text was carried out using NVivo 12. In the quantitative analysis, univariable and multivariable ordinal logistic models were used to examine the potential factors for preference among students in higher education. A total of 380 Nursing and Social Science students responded to the survey. Approximately 77% of students perceived non-invigilated assessments to be less stressful compared to invigilated exams. Age, course of study, stage of studies, and number of units enrolled per semester were identified as significant drivers for students’ perceived preference for non-invigilated assessments. There was an inverse relationship between the perception of stress associated with invigilated exams and the age of students. For instance, students aged between 18-24 were 5 times more likely to prefer non-invigilated exams compared to those aged 55 or more. Comparatively, students in early stages of studies had higher preference for non-invigilated assessments. However, there was a preference reversal for students enrolled in 2 or less units per semester. Social sciences students were two times more likely to prefer non-invigilated examinations to invigilated examinations compared to nursing students. The findings reinforce the use of alternative assessments in higher education as a mitigating agency to lessen the mental health burden of tertiary students in post COVID-19 era.

**Supplementary Information:**

The online version contains supplementary material available at 10.1007/s10805-023-09472-w.

## Introduction

The COVID-19 pandemic has heightened mental health difficulties across all spheres of life. More specifically, in higher education, the rapid changes in the mode of teaching, learning and assessments have affected course structure, loss of work integrated learning and conduct of examinations (Alsaady et al., 2020; Rasmussen et al., 2021). Mental health difficulties can impact students’ overall academic outcomes and wellbeing (Rasmussen et al., 2021; Ribeiro et al., 2018). As assessments are integral part of higher education, higher education institutions made rapid changes to assessment modalities—from traditional examinations to other innovative forms—which contributed to stress and test anxiety among students (Almossa, 2021; Alsaady et al., 2020).

At the peak of the pandemic, traditional summative assessments such as invigilated examinations became unpopular due to COVID-19 restrictions on public gathering. To remain competitive, innovative assessments designs were introduced in higher education (Fuller et al., 2020). Amid the pandemic, a variety of summative assessment formats (collectively known as alternative assessments) became common in Australian Universities. Alternative assessments such as take-home open book non-proctored assessment, remote viva, and video practical demonstrations replaced the traditional invigilated examinations.

Assessment related stress is a common phenomenon for both students and staff with educational researchers constantly searching for strategies to undertake authentic assessments while maintaining students’ wellbeing (Bengtsson, 2019; Jones et al., 2021). It is an established fact that invigilated examinations cause undue stress and do not represent real world case scenarios (Bengtsson, 2019; Jones et al., 2021). Summative assessment related mental health difficulties such as test anxiety is common among undergraduate students (Alsaady et al., 2020; Hamzah et al., 2019; Nsor-Ambala, 2020). On average, it affects 70% of undergraduate students (Hamzah et al. (2018); Numan & Hasan, 2017;. The negative consequence of test anxiety include loss of memory, misreading questions, psychosomatic disorders (headaches and gastrointestinal disturbances) resulting in adverse effects on learning and test performance (Alsaady et al., 2020; Hamzah et al., 2019). Despite this, when test anxiety is perceived as a positive emotion, students can adapt and cope, leading to improved memory, motivation, concentration levels and academic performance (Brady et al., 2018; Fischer et al., 2016; Numan & Hasan, 2017). Even so, prolonged anxiety can intensify and progress into panic attacks and overall progressive mental health decline (Alsaady et al., 2020; Hamzah et al., 2019).

Test anxiety among students have been studied in both non-invigilated online and traditional modes of examinations (Lowe, 2019; Alibak et al., 2019; Mastour et al., 2021). While the literature is not concrete on which type of assessment—online or traditional—causes more anxiety, there is evidence to suggest that students experience a certain level of test anxiety with all forms of assessments (Harley et al., 2021; Keimer et al., 2022). Proponents of online assessment report that it reduces overall test anxiety (Alderbashi, 2021). Similarly, Akulwar-Tajane et al. (2021) found that among physiotherapy students, open-book assessments during the COVID-19 pandemic reduced students’ stress levels. However, there is paucity of evidence on alternative assessment and mental health wellbeing of university students, particularly Social Science and Nursing students.

Compared to other students, nursing students have been reported to have experienced higher levels of mental health difficulties (stress and depression) during the COVID-19 pandemic (Rasmussen et al., 2021; Thomas, 2021). The mental health difficulties were attributed to the cancellation of their clinical placements and the abrupt transition to online learning and assessments during the pandemic (Rasmussen et al., 2021). Many nursing schools resulted to innovative and alternative ways of assessing clinical skills and theoretical knowledge which added additional layer of stress for nursing students (Hayes & McCauley, 2021; Ulenaers et al., 2021). However, no study has explored the possible impact of these innovative alternative assessments on the mental health vulnerability of nursing students. Additionally, no study has evaluated the possible nuances of alternative assessments across different disciplines in higher education and their perceived impact on students’ mental wellbeing.

Bengtsson (2019) study suggests open book non-invigilated examinations increase critical thinking, knowledge and learning but they require more time to complete. They are perceived to be less stressful, reduce test anxiety and generally improve students’ grades (Teodorczuk et al., 2018). Although there is ongoing debate on the authenticity of alternative assessments (Fuller et al., 2020; Reisenwitz, 2020), when designed well, they can provide the same (if not better) outcomes as invigilated exams (Cluskey Jr et al., 2011).

Years after the initial COVID-19 disruption, less summative examinations are invigilated (Cutler, 2021) with a drastic shift to non-invigilated alternative assessments. In most universities, alternative assessments are commonly used for summative assessments (Fuller et al., 2020). However, less is known about how these assessments impact on students’ mental health wellbeing. Thus, after successfully completing the first round of alternative assessment in July 2020 at an Australian public university, we sought to describe nursing and social science students’ perceptions of the impact of alternative assessments on their mental health during the COVID-19 pandemic and to explore how these may influence the future of assessments in higher education.

## Methods

### Study Design

The study utilised a retrospective cross-sectional design with closed and open-ended questions administered to undergraduate and postgraduate nursing and social science.

### Survey Tool Development

There is a plethora of evidence about test anxiety and validated tools that measure test anxiety for traditional examinations; more recent studies combined tools to include online computerised exams (Alibak et al., 2019; Arora et al., 2021; Cazan & Indreica, 2014; Stowell & Bennett, 2010). However, many of these tools are focused on online examinations and are academic perspective and were not altered due to a pandemic (Davies, 2015; Alderbashi, 2021; Harley et al., 2021). Whilst the team acknowledges relevant studies and tools, the focus of this paper is the impact of alternative assessments (not examinations) on students’ mental health during the COVID-19 pandemic and to explore how the various methods may influence the future of assessments in higher education.

Additionally, as COVID-19 is a novel disease, there were no validated tools to assess the alternative assessments developed due to the COVID-19 restrictions. Therefore, a self-reported online questionnaire was developed by the research team based on evidence on higher education teaching, assessment, academic integrity, and emotional wellbeing behaviours (Adesile et al., 2016; McCabe & Trevino, 1993; Ramdani, 2018). The face validity of the instrument was assessed through multiple consultation with stakeholders—experts and students. First, using evidence, a draft survey tool was developed. It then went through a series of consultations with the research team, two experts (Associate Deans of Teaching and Learning from the two schools) in higher education teaching and learning, and assessments practices. The initial survey questions were then revised based on expert feedback for face validity. Following this, the survey tool was piloted with undergraduate and postgraduate students for clarity and appropriateness of the items to elicit the responses on students’ perception of alternative assessment. The feedback was used to update and finalise the survey before data collection.

The survey consisted of 22 items expected to be completed within 15–20 min. The self-reported survey included a 5-point Likert scale which compared proctored/invigilated examinations with non-proctored alternative assessments and open-ended questions to provide further information on the Likert scale responses. This paper presents an aspect of a larger study that investigated a few silo components of students’ perception with alternative assessments. Additionally, a 5-point Likert scale on the level of stress associated with proctored versus non-proctored alternative assessment was also included to assess the level of test related mental health difficulties. We collected demographic information such as age and level of education, programme/course of study and number of subjects/units enrolled in the preceding semester. These demographic information were considered typical in exploring any possible diversity thereof. Additionally, a unique opportunity with alternative assessments is flexibility of scheduling the duration and identify whether time had any influence on students’ mental health difficulties.

The study explored the perceptions of students regarding their stress levels when undertaking the alternative assessments. The survey (Appendix A) was piloted with a small number of undergraduate students to ensure the questions were easy to understand and were appropriate at undergraduate level of comprehension (Bowden et al., 2002).

Convenience sampling was applied and all undergraduate and postgraduate students across two schools (School of Nursing and Midwifery and School of Arts and Humanities) invited to participate in the survey. Students who completed the pilot survey were excluded from the main study.

### Participants

Students from two schools and across different stages within their courses in an Australian public university took part in the study. Participants had previously experienced invigilated examination at either secondary school or university before the COVID-19 pandemic. Having experienced both invigilated examinations and alternative assessments, students were invited to compare the mental health difficulties associated with the two types of assessments. Data was collected from August to December 2020 for both undergraduate and postgraduate domestic and international students.

### Ethical Consideration and Data Collection

Ethics approval was obtained from the human research ethics committee of the university 2020–01533-ADAMA where the study was conducted. Informed consent (a forced response) and information sheets were embedded in the Qualtrics survey. All students provided consent prior to commencing the study.

### Data Analysis

#### Quantitative

The quantitative segment analysed the relationship between demographics (age, course, level and stage of study and number of units enrolled) and mental wellbeing measured by levels of stress. Analyses were carried out in IBM SPSS version 26. Descriptive analysis (frequencies and percentages) highlighting the distributions across levels of the demographics for the measure was undertaken. We fitted univariable and multivariable generalised ordinal logistic regression models to quantify the magnitude of effects of potential drivers of tertiary students’ preference or otherwise for non-invigilated assessments in stress alleviation. Crude odds ratio (OR) and adjusted odds ratio (AOR) were also reported. Missing data for each variable was identified and removed from the analysis. Two-sided p-values < 0.05 of a 95% confidence interval (CI) was considered significant.

#### Qualitative

An open-ended question was included to provide further depth and allow participants to share their experience of stress associated with non-invigilated alternative assessments. Participants responded to the specific open-ended question *“I prefer invigilated exams to the alternative assessment, please explain”.* Qualitative data were analysed inductively by two authors. NViVo 12 was used to support the analysis. Data analysis followed the six stages step-by-step approach by Braun and Clarke (2006). Analysis followed in-depth familiarisation of self with the data, followed by coding and theme generation with corresponding quotations from the data.

## Results

### Quantitative Finding

Of the 380 study participants, 302 (79.4%) responded to the question about stress, the majority aged between 18–24 (n = 112; 29.5%), closely followed by 25–34 age group (n = 94; 24.7%). More than half of the participants were enrolled in a nursing course (n = 209; 55%); 83.9% were undergraduate students (n = 319), with most of them in their second year of study (n = 130; 34.2%) and enrolled into at least four units per semester (n = 113; 29.7%). The commonly reported timeframe assigned to complete non-invigilated exams was 24 h (n = 183; 48.2%), followed by assessments completed within 2 to 4 h (n = 147; 38.7%). Descriptive statistics, mean, standard deviation and total score for the appropriate variables is tabled (Appendix B). Frequencies and correlations are available in both Table 1 and 2. The distribution of the frequency of the 5-scale Likert responses to the proposition that invigilated exams are more stressful than non-invigilated exams across the demographic features are also presented in Table 1. Approximately 77% of participants perceived non-invigilated assessments were less stressful compared to invigilated assessments (Table 1).

**Table 1:** Background characteristics of study sample and frequencies of responses to the comparative perceived stress associated with invigilated exams and non-invigilated exams

		**Invigilated exams are more stressful than non-invigilated exams**					
**Variable**	**N (%)**	**SD**	**D**	**NAD**	**A**	**SA **	**NRR**
		**N (%)**	**N (%)**	**N (%)**	N (%)	N (%)	
*Age*							
18–24	112 (29.5)	2 (0.52)	3 (0.79)	4 (1.05)	15 (3.95)	74 (19.47)	15 (3.95)
25–34	94 (24.7)	4 (1.05)	7 (1.84)	8 (2.29)	14 (3.68)	49 (12.89)	12 (3.16)
35–44	73 (19.2)	4 (1.05)	10 (2.63)	10 (2.63)	8 (2.29)	36 (9.47)	5 (1.32)
45–54	40 (10.5)	5 (1.32)	5 (1.32)	2 (0.52)	7 (1.84)	18 (4.74)	4 (1.05)
55 & above	11 (2.9)	1 (0.26)	1 (0.26)	2 (0.52)	2 (0.52)	3 (0.79)	2 (0.52)
(Missing)	50 (13.2)						
*Course of study*							
Social Sciences	129 (33.9)	4 (1.05)	15 (3.95)	9 (2.37)	15 (3.95)	79 (20.79)	8 (2.29)
Nursing	209 (55.0)	12 (3.16)	13 (3.42)	18 (4.74)	32 (8.42)	105 (27.63)	38 (10.00)
(Missing)	42 (11.1)						
*Level of study*							
Undergraduate	319 (83.9)	15 (3.95)	27 (7.11)	24 (6.32)	44 (11.58)	176 (46.32)	41 (10.79)
Postgraduate	16 (4.2)	1 (0.26)	1 (0.26)	3 (0.79)	3 (0.79)	6 (1.58)	2 (0.52)
(Missing)	45 (11.8)						
*Stage of study*							
Year 1	111 (29.2)	4 (1.05)	5 (1.32)	9 (2.37)	13 (3.42)	69 (18.16)	14 (3.68)
Year 2	130 (34.2)	4 (1.05)	12 (3.16)	10 (2.63)	17 (4.47)	73 (19.21)	20 (5.26)
Year 3	75 (19.7)	7 (1.84)	9 (2.37)	5 (1.32)	12 (3.16)	33 (8.68)	10 (2.63)
Postgrad	22 (5.8)	1 (0.26)	2 (0.52)	3 (0.79)	5 (1.32)	9 (2.37)	2 (0.52)
(Missing)	42 (11.1)						
*Number of units enrolled*							
1	62 (16.3)	4 (1.05)	9 (2.37)	7 (1.84)	9 (2.37)	28 (7.37)	5 (1.32)
2	80 (21.1)	2 (0.52)	8 (2.29)	9 (2.37)	15 (3.95)	39 (10.26)	8 (2.29)
3	81 (21.3)	2 (0.52)	7 (1.84)	6 (1.58)	6 (1.58)	50 (13.16)	14 (3.68)
4 or more	113 (29.7)	8 (2.29)	4 (1.05)	5 (1.32)	17 (4.47)	67 (17.63)	17 (4.47)
(Missing)	44 (11.6)						
*Duration of alternative assessment*							
2–4 h	147 (38.7)	7 (1.84)	7 (1.84)	11 (2.89)	22 (5.79)	84 (22.11)	16 (4.21)
24 h	183 (48.2)	8 (2.29)	17 (4.47)	11 (2.89)	24 (6.32)	107 (28.16)	16 (4.21)
48 h	48 (12.6)	11 (2.89)	3 (0.79)	1 (0.26)	6 (1.58)	25 (6.58)	8 (2.29)
1–2 week(s)	19 (5.0)	2 (0.52)	0 (0)	2 (0.52)	2 (0.52)	11 (2.89)	2 (0.52)
Other	61 (16.1)	1 (0.26)	7 (1.84)	4 (1.05)	8 (2.29)	34 (8.95)	7 (1.84)

*SD* strongly disagree, *D* disagree, *NAD* Neither agree nor disagree, *A* Agree, *SA* strongly agree, *NRR* No-response rate

**Table 2:** Univariable and multivariable analyses of demographic risk factors associated with the level of agreeableness to the preference of alternative assessment in managing stress

**Variables**	**Univariable analysis**			**Multivariable analysis**		
	**Est. (SE)**	**OR (95% CI)**	**p-value**	**Est. (SE)**	**AOR (95% CI)**	**p-value**
***Age***						
18 – 24	1.696 (0.626)	5.454 [1.600, 18.596]	**0.007**	1.964 (0.696)	7.125 [1.822, 27.856]	**0.005**
25 – 34	0.929 (0.619)	2.532 [0.753, 8.519]	0.133	1.172 (0.672)	3.227 [0.865, 12.043]	*0.081*
35 – 44	0.557 {0.623)	1.745 [0.515, 5.920]	0.371	0.686 (0.683)	1.986 [0.521, 7.569]	0.315
45 – 54	0.371 (0.658)	1.449 [0.399, 5.263]	0.573	0.373 (0.711)	1.452 [0.361, 5.849]	0.600
55 and above	0	1	**-**	0	1	**-**
***Course***						
Social Sciences	0.211 (0.235)	1.235 [0.780, 1.957]	0.368	0.736 (0.298)	2.087 (1.163, 3.746]	**0.014**
Nursing	0	1	**-**	0	1	**-**
***Level***						
Undergraduate	0.593 (0.487)	1.810 [0.696, 4.704]	0.224−	0.167 (0.961)	0.846 [0.129, 5.559]	0.862
Postgraduate	0	1	-	0	1	-
***Stage***						
Year 1	0.830 (0.452)	2.294 [0.947, 5.559]	*0.066*	0.623 (0.846)	1.865 [0.356, 9.782]	0.461
Year 2	0.554 (0.440)	1.739 [0.734, 4.119]	0.208	0.554 (0.836)	1.740 (0.338, 8.956]	0.508
Year 3	–0.023 (0.461)	0.978 [0.396, 2.413]	0.961	–0.154 (0.833)	0.857 [0.167, 4.391]	0.854
Postgraduate	0	1	-	0	1	-
***Number of units per semester***						
1	–0.748 (0.323)	0.473 [0.252, 0.891]	**0.020**	–0.245 (0.399)	0.783 [0.358, 1.710]	0.539
2	–0.453 (0.301)	0.636 [0.353, 1.146]	0.132	–0.047 (0.355)	0.954 [0.476, 1.913]	0.894
3	0.128 (0.329)	1.137 [0.596, 2.168]	0.697	0.255 (0.364)	1.291 [0.633, 2.632]	0.483
4 or more	0	1	-	0	1	-
***Duration of alternative assessment***						
2–4 h	0.293 (0.231)	1.341 [0.852, 2.109]	0.205	0.388 (0.334)	1.474 [0.767, 2.835]	0.244
24 h	0.251 (0.228)	1.285 [0.822, 2.009]	0.271	0.372 (0.332)	1.451 [0.756, 2.783]	0.263
48 h	–0.028 (0.345)	0.972 [0.495, 1.911]	0.935	0.037 (0.388)	1.037 [0.485, 2.219]	0.925
1 week	0.315 (0.859)	1.370 [0.255, 7.375]	0.714	0.305 (0.937)	1.356 [0.216, 8.509]	0.745
2 weeks	–0.013 (0.626)	0.987 [0.290, 3.364]	0.983	0.382 (0.824)	1.465 [0.291, 7.371]	0.643
Other	0.112 (0.300)	1.118 [0.621, 2.012]	0.710	0.548 (0.401)	1.730 [0.788, 3.797]	0.172

Bold p-values denote significant association at 5% level of significance; Categories with 0 estimates were used as reference levels in the modelling

*Est* parameter estimate, *SE* Standard error, *OR* Odds ratio, *AOR* Adjusted Odds ratio, *CI* confidence interval

We further quantified the magnitude of effects of the demographic features in the evaluation their associations in the perceived preference for non-invigilated assessments over invigilated assessments in relation to exams related stress in both univariable and multivariable analyses (Table 2).

In the univariable analysis (i.e., each demographic feature was expressed as a function of perceived responses to the proposed preference question), age (*p* < *0.001*), stage of study (*p* < *0.031*) and number of units per semester (*p* < *0.037*) were identified as the significant contributing factors to the perceived preference for non-invigilated exams. Based on the reported odds, it can be inferred that the preference for non-invigilated exams in stress management decreases with age. For instance, students aged between 18–24 are 5 times more likely to prefer non-invigilated exams compared to matured students aged 55 or more. The reported odds (OR) of preference decreased at the increasing age categories (i.e., compared to students aged 55 or more, students within the age bracket of 25–34 were 3 times more likely in their preference for non-invigilated exams, those in 35–44 bracket were 1.7 times more likely, whilst those in 45–54 bracket were 1.4 times more likely in their preference). Similarly, students in lower stages of studies had higher preference for non-invigilated exams. For examples, compared to third year students, first and second year students were 2.2 and 1.7 times more likely to prefer non-invigilated exams to invigilated exams respectively. Students enrolled in 2 or less units were less likely to prefer non-invigilated exams to invigilated exams compared to students enrolled in 3 or more units.

In the multivariable analysis (i.e., all demographic features were jointly expressed as a function of perceived responses to the proposed preference question). Age and course of study were identified as significant contributors in perceived preference for non-invigilated exams over invigilated exams. Like the univariate results, there was a decreasing preference for non-invigilated exams with increase in age. The adjusted odds (AOR) indicated that compared to students aged 55 or more, those in the following brackets, 18–24, 25–34, 35–44 and 45–54 were 7, 3, 1.9 and 1.4 times more likely to prefer non-invigilated exams to invigilated exams respectively. In relation to course of study, social sciences students were 2 times more likely to prefer non-invigilated exams to invigilated exams compared to nursing students. The trends in preferences observed in the multivariable results for stage of study and number of units per semester aligned to their respective univariate results.

### Qualitative Findings

#### Emotional Response to Proctored and Non-Proctored Assessments

The open-ended questions generated text that reiterated the findings from the quantitative data. In total, 277 responses related to the comparison of invigilated examination and alternative open-book assessment were obtained. The main theme from the qualitative data is emotional response to the proctored and non-proctored assessment.

Majority of students stated that alternative assessment reduced their anxiety and stress levels. They explained *“…spiking anxiety during examinations”*; “*invigilated exams are stressful”*; “*invigilated exams give me anxiety”, invigilated exams are anxiety-inducing for students,* and *psychologically uncomfortable for some;* however, alternative assessments were reported as being *“much less stressful and intimidating than normal exams*” and “*I felt less stress and more accomplished after alternative assessments”*.

For majority of students, test anxiety and stress emanated from their inability to rote learn, presence of invigilators who can, sometimes, be a form of distraction; accessing the university (driving from far and parking); examination halls and personal responsibilities such as childcare. Thus, having to do their alternative assessments in the comfort of their homes reduced their anxiety and stress. In addition, students stated that alternative assessments expanded on their learning. Such views were captured in the following quotes:

*“I get very anxious with invigilated exams which impacts my ability to rationally answer the exam questions”*

*“It [alternative assessment] takes away the stress and nerves when you can work from home, not travel to campus, look for parking, queue to get into room, deal with rude invigilators etc all before you sit down to take the exam.”*

*“Invigilated exams more stress to me than alternative assessment because I panic in an exam situation if I feel like I do not have control over the situation and I am trapped in a room with people for many hours. And add the stress from completing the exam in time and getting the right answers”*

On the other hand, few students felt that alternative assessments increased their test anxiety as the duration for some assessments was too long and they could not switch off until the assessment due date. For example, given they had such a long time (an average of 24 h) to submit their assessment, students were not sure if what they had written was good enough, resulting in increased test anxiety. Despite the long duration, students acknowledged that the nature of the assessments enabled them to experience high order learning skills such as critical thinking and problem-solving skills. Others also attributed test stress to the level of quality expected as they lamented:

*“More learning planning for alternative assessment, more critical research, but way more stressful. Quality expectations are higher with alternative assessment, which adds to the stress.”*

Some students found alternative assessment more stressful than invigilated assessment as they perceive alternative assessments are advantageous for students with high Information Technology (IT) skills and may not be equitable for students with less skills or unstable internet:

*“After having done invigilated exams for 3 years, I have never before ever run out of time and failed to complete a question until these online assessments were introduced. I believe they are unfair, inequitable and biased towards those with more IT experience, better typing skills, and more reliable internet. The idea of my internet crashing mid-test (in a short, timed assessment) was an extra stress I really didn't need.”*

## Discussion

Before the COVID-19 pandemic, higher education institutions relied on proctored/invigilated assessments. Although this type of assessment has been criticised, it has remained the most common approach. However, with COVID-19 restrictions, traditional invigilated examinations gave way to alternative assessments in higher education across the globe. In this study, we analysed both quantitative and free-text data from 380 undergraduate and postgraduate nursing and social science students. Majority of research participants found online non-invigilated alternative assessments to be less stressful compared to traditional invigilated/proctored assessments. Participants reported that alternative assessments are associated with deep learning due to reduction in test anxiety and stress.

The findings showed significant association between age and stress; self-reported stress was relatively lower among students within the age groups 18–24 and 25–34 compared to those aged 35 and above. In a related study, Dawood et al. (2016) investigated the impact of test anxiety on cumulative assessment grades among undergraduate nursing students and reported decreasing test anxiety with increasing age. The high levels of self-reported stress among mature-aged students may be due to other confounding factors, such as family commitments and low levels of IT skills, which could, in part, impact negatively on their academic performance compared to younger students. To ensure equity, mature-aged students with IT challenges should be given extra IT support prior to the commencement of online alternative assessments. It was found that undergraduate students reported higher levels of test anxiety and stress than postgraduate students. This finding is similar to that of Numan and Hasan (2017) where they reported high levels of test anxiety among undergraduate students.

Students perceived alternative assessments to reduce their stress levels compared to invigilated examination. Though a certain amount of stress is good for optimum performance, there is evidence to suggest that too much stress can affect the academic and/or cognitive performance of students (Alsaady et al., 2020; Nsor-Ambala, 2020). For years, academics have been researching into alternative ways to manage test anxiety and stress among university students. Strategies such as cognitive behavioural therapy, coping skills, massage, relaxation, and imagery techniques have proven effective (Yusufov et al., 2019). However, our study has shown that alternative assessments such as open book take home assessments could be considered an additional strategy for reducing examination induced stress and anxiety. Among Australian university students, stress and anxiety levels continue to increase as a result of the COVID-19, thus, it is imperative for academics to start planning for support strategies including, assessment modalities that support the mental wellbeing of students (Kochuvilayil et al., 2021).

The current study revealed that in comparison with social science students, nursing students scored low in answering ‘*YES’* to the question: ‘*Is alternative assessment less stressful than invigilated assessment?’* This observation could be because nursing students often undertake VIVAS, which are known to be more stressful than written assessments (Rajanayagam, 2020). This finding supports Kochuvilayil et al. (2021), study, which found higher levels of stress among Australian nursing students than nursing students in India during the peak of the COVID-19 pandemic. To support the fragile mental health of nursing students (Rasmussen et al., 2021; Rosenthal et al., 2021), alternative assessments may become the gold standard for during and beyond the COVID-19 pandemic. Perhaps, it is time to change VIVAS to help reduce stress and develop a different format to elicit the same information.

With COVID-19 came students’ mental health vulnerability and the call for further action to mitigate the mental health burden of students (Grubic et al., 2020). As the world come to terms with the reality of living with COVID-19, non-invigilated assessment designs should consider both alternative types of assessments and preservation of students’ mental wellbeing.

Invigilated assessment increased the self-reported students’ stress and did not encourage critical thinking but made them feel trapped as they could not perform well in the presence of ‘Big Brother’. Duration was not significant in students’ perceived preference of non-invigilated assessments. However, with alternative assessments, students were able to perform at their best— stress reduction, think critically— which resulted in high performance. These findings were illustrated in the qualitative data and confirm the work of Sohail et al. (2020) which reported that stress has negative impact on the academic performance of students, but in contrast to the work of Dawood et al. (2016), they found no relationship between test anxiety and academic performance. Despite either opinion, as future work skills are moving from task-orientation to critical and problem-solving orientation, assessment designs should be authentic and reflect current industry practices.

## Limitations and Future Research

The study is limited by the lack of validated tool to assess test anxiety; however, Roos et al. (2021) assert that self-reported test anxiety are positively related to the physiological characteristics of test anxiety and can be considered equally effective in assessing test anxiety. The construct validity of the data collection tool was lacking; future research should focus on assessing the construct validity of the current tool for alternative assessments. As the study involved less than 400 participants from two disciplines in a single university setting, generalisation of the findings should be done with caution. Future studies should broaden the scope to include other universities and disciplines and a larger sample.

## Conclusion

Assessment is an integral part of higher education. However, due to the COVID-19 restrictions, educational institutions have had to adapt new and alternative ways of assessing students. Our study revealed that alternative assessments (non-invigilated and take-home assessments) were perceived to be effective in promoting mental wellbeing of students compared to traditional invigilated examinations. Moreso, students reported feeling relaxed due to the flexibility of assessment timing (can sit for the assessment anytime within a given period) and less mental health difficulties with alternative assessments. COVID-19 has provided the impetus for education institutions to review their students’ assessments. Our findings showed that alternative assessments enhance students’ mental wellbeing, which is fundamental to the learning process and overall student success. These findings have implications for academics, teaching and learning designers including deans of teaching and learning, to ensure that mental health considerations are made when designing assessments. It is recommended that higher education assessment policies consider alternative assessments as a viable option to examine holistic students’ learning.

## Supplementary Information

Below is the link to the electronic supplementary material.

Supplementary file1 (DOCX 16 KB)

## Data Availability

The dataset generated during the study are available from the corresponding author on reasonable request.
