# Supplementary material for: Perceived Nexus Between Non-Invigilated Summative Assessment and Mental Health Difficulties: A Cross Sectional Studies
Source: J Acad Ethics. 2023 Mar 31:1–15. Online ahead of print. doi: 10.1007/s10805-023-09472-w (PMC10063941; doi:10.1007/s10805-023-09472-w)
Supplement: Supplementary file 1 — Supplementary file1 (DOCX 16 KB) [file 10805_2023_9472_MOESM1_ESM.docx]

**Appendix A**

Survey for alternative assessments and implications for stress

Start of Block: Consent form

Q1 **CONSENT**

- Yes
- No

Skip To: End of Survey = No

End of Block: Information letter and consent form

Start of Block: Demographic data

Q2 How old are you?

▼ 18-24 (1) ... 75 and above (10)

Q3. Which course are you currently enrolled in?

- ________________________________________________

Q4. Are you enrolled in an undergraduate course or postgraduate course?

- ________________________________________________

Q5. Which stage of your course are you enrolled in?

- ________________________________________________

Q6. In 2020 Semester 1, how many units did you undertake a take-home alternative assessment?

- 1 (1)
- 2 (2)
- 3 (3)
- 4 (4)
- 5 or more (5)

Q7. Please indicate the timeframe you were allocated to complete the alternative assessment, once opened?

- 2-4 hours (1)
- 24 hours (2)
- 48 hours (3)
- 1 week (4)
- 2 weeks (5)
- Other please specify

End of Block: Demographic data

Q8. Compared to an invigilated examination, to what extend do you agree or disagree with the following statement?

|  | Strongly agree (1) | Agree (2) | Neither agree nor disagree (3) | Disagree (4) | Strongly disagree (5) |
| --- | --- | --- | --- | --- | --- |
| Invigilated exams cause more stress than an alternative assessment  (5) |  |  |  |  |  |

Q9. What are your overall thoughts/feelings about the alternative assessment?

________________________________________________________________
